# Impact of ZnO NPs on photosynthesis in rice leaves plants grown in saline-sodic soil

**DOI:** 10.1038/s41598-024-66935-9

**Published:** 2024-07-14

**Authors:** Kun Dang, Yuxin Wang, Hao Tian, Jingjing Bai, Xiyuan Cheng, Liying Guo, Qiang Zhang, Yanqiu Geng, Xiwen Shao

**Affiliations:** 1 Agronomy College, Jilin Agricultural University, Changchun, 130118 China; 2 Key Laboratory of Germplasm Innovation and Physiological Ecology of Coldland Grain Crops, Ministry of Education, Harbin, 150000 China; 3 Jilin Provincial Laboratory of Crop Germplasm Resources, Changchun, 130118 China

**Keywords:** Rice, Saline-sodic stress, ZnO NPs, Chlorophyll fluorescence, Photosynthesis, Plant stress responses

## Abstract

Saline-sodic stress restricts the absorption of zinc by rice, consequently impacting the photosynthesis process of rice plants. In this experiment, Landrace 9 was selected as the test material and the potting method was employed to investigate the influence of ZnO nanoparticles (ZnO NPs) on zinc absorption and chlorophyll fluorescence in rice grown in saline-sodic land. The research findings demonstrate that the application of ZnO NPs proves to be more advantageous for the growth of rice in saline-sodic soil. Notably, the application of ZnO NPs significantly decreases the levels of Na^+^ and MDA in rice leaves in saline-sodic soil, while increasing the levels of K^+^ and Zn^2+^. Additionally, ZnO NPs enhances the content of chloroplast pigments, specific energy flux, quantum yield, and the performance of active PSII reaction center (*PI*_ABS_) in rice leaves under saline-sodic stress. Furthermore, the relative variable fluorescence (*W*_K_ and *V*_J_) and quantum energy dissipation rate (*φ*_Do_) of rice are also reduced. Therefore, the addition of ZnO NPs enhances the transfer of electrons and energy within the rice photosystem when subjected to saline-sodic stress. This promotes photosynthesis in rice plants growing in saline-sodic land, increasing their resistance to saline-sodic stress and ultimately facilitating their growth and development.

## Introduction

Soil salinization is a global environmental and agricultural catastrophe, and it is a major abiotic stress factor that hinders the normal growth and development of crops^1^. The salinized soil in Songnen Plain, which is one of the key grain-producing regions in China, significantly restricts crop yield^2^. The impact of salinized soil on plant growth primarily manifests through osmotic imbalance, ionic toxicity, and high pH stress^3^. With the increasing salinization of land, numerous physical, chemical, and biological methods have been developed to mitigate the damage caused by saline-sodic stress on crops^4^. These methods primarily aim to enhance the biochemical pathway of saline-sodic tolerance, resulting in a synergistic effect. Research has demonstrated that regulating ion absorption can alleviate ion toxicity in crops and preserve the integrity of cell membranes, thereby providing a stable reaction site for crucial physiological processes such as photosynthesis^5,6^. The study also found that as the concentration of saline-sodic increased, the leaf water potential and evaporation rate of crops under saline-sodic stress decreased significantly^7^. This suggests that adjusting the synthesis of compatible solutes could alleviate the osmotic stress of crops^8^. Furthermore, changing the photosynthetic pathway could increase crop yield^9^. Recent studies have shown that the addition of exogenous substances such as hormones and metal elements has a positive effect on improving crop growth in saline-sodic land^10^. This indicates that the addition of foreign aid substances can effectively promote crop growth in such conditions.

Zinc is an essential trace element for plant growth and plays a crucial role in plant disease resistance, photosynthesis, cell membrane integrity, protein synthesis, pollen formation, and enhancing levels of antioxidant enzymes and chlorophyll in plant tissues^11^. However, the salinized soil in Songnen Plain, one of the main grain-producing areas in China, severely hinders the absorption of nutrients like zinc by crops, thereby affecting their growth and development^12^. Studies have demonstrated that zinc can increase the concentration of macronutrients and micronutrients in rice under saline-sodic stress while reducing the concentration of Na^+^^13^. Zinc not only reduced the accumulation of soluble sugar and starch in rice leaves, but also alleviated the feedback inhibition of photosynthesis and increased the net photosynthetic rate^14^. Additionally, it promoted the transport of assimilation products to the underground part, providing more energy for rice root growth under saline-sodic stress. This improved the ability to resist saline-sodic stress, thereby promoting the growth and development of rice. However, in the Songnen plain rice area with high pH, the available zinc content decreases rapidly with increasing pH, especially in soil containing bicarbonate^15^. This limitation severely hampers the absorption and utilization of zinc by crops. Nanotechnology is considered a promising field in agricultural science and plays a significant role in revolutionizing agriculture and food production by effectively managing soil nutrients^16,17^. In recent years, zinc nanoparticles have gained popularity for their ability to enhance zinc absorption in plants, thereby improving the effectiveness of zinc fertilizers^18^. The use of nanoparticles as fertilizers has the potential to benefit agriculture. These particles, typically 100 nm in size, have a spherical or faceted shape and possess a specific surface area of about 30–50 m^2^ g^−1^^19^. As the specific surface area increases, their catalytic properties improve. Additionally, nanoparticles exhibit high dispersibility and absorbency^20^. Nano-fertilizer has the potential to enhance the absorption of nutrients by plants, leading to increased yield and nutrient content in edible plant parts^21^. Research has demonstrated that the use of ZnO NPs can enhance crop growth by modulating the generation of reactive oxygen species like superoxide and hydroxide anion^22^. ZnO NPs have been found to enhance the concentrations of chlorophyll, carotenoid, protein, and antioxidant enzymes in rice plants^23^. At the same time, due to its highly conductive, non-toxic, easy to obtain, eco-friendly, cost-effective, and inert properties, zinc in nano form shows great potential for enhancing plant growth and aiding in adaptation to environmental stress. Research has demonstrated that ZnO-NPs have the potential to enhance plant growth and increase the accumulation of antioxidants, penetrants, and secondary metabolites. This can aid plants in coping with the detrimental effects of salt stress^24^. Additionally, studies have indicated that the incorporation of ZnO-NPs can alleviate salt stress in rice by upregulating the antioxidant system. However, ZnO NPs have been shown to have toxic effects on plants. Lin et al. discovered that a concentration of 2000 mg L^−1^ of ZnO NPs hindered seed germination and root elongation in various plant species, including radish, rape, ryegrass, lettuce, corn, and cucumber. This indicates that the toxicity is attributed to the particles themselves^25^. In contrast, Stampoulis et al. found that a concentration of 1000 mg L^−1^ of ZnO NPs or large-particle ZnO in a hydroponic solution did not impact seed germination, root elongation, or biomass of zucchini^26^. On the other hand, Mahajan observed that low concentrations of ZnO NPs can actually stimulate the growth of mung beans, while high concentrations inhibit their growth^27^. Therefore, factors such as the specific nanomaterial, plant species tested, concentration, and particle size are crucial in determining the phytotoxicity of nanoparticles.

Therefore, it is necessary to investigate whether the application of nano-zinc oxide (ZnO NPs) can effectively promote the absorption and utilization of zinc by rice in saline-sodic soil, especially when compared to traditional ZnSO_4_. This study focuses on Changbai 9, the main rice variety grown in saline-sodic areas of Jilin Province. The analysis of chlorophyll fluorescence using chlorophyll fluorescence and 820 nm technology was conducted to evaluate the effects of saline-sodic stress^28^. The study also examines the impact of ZnO NPs and ZnSO_4_ on chlorophyll fluorescence and electron transport in rice leaves. The study hypothesizes that ZnO NPs is more beneficial for zinc absorption in saline-sodic rice areas and that it has a greater effect on chlorophyll fluorescence and electron transfer in rice.

## Materials and methods

### Experimental site

**Figure 1:**
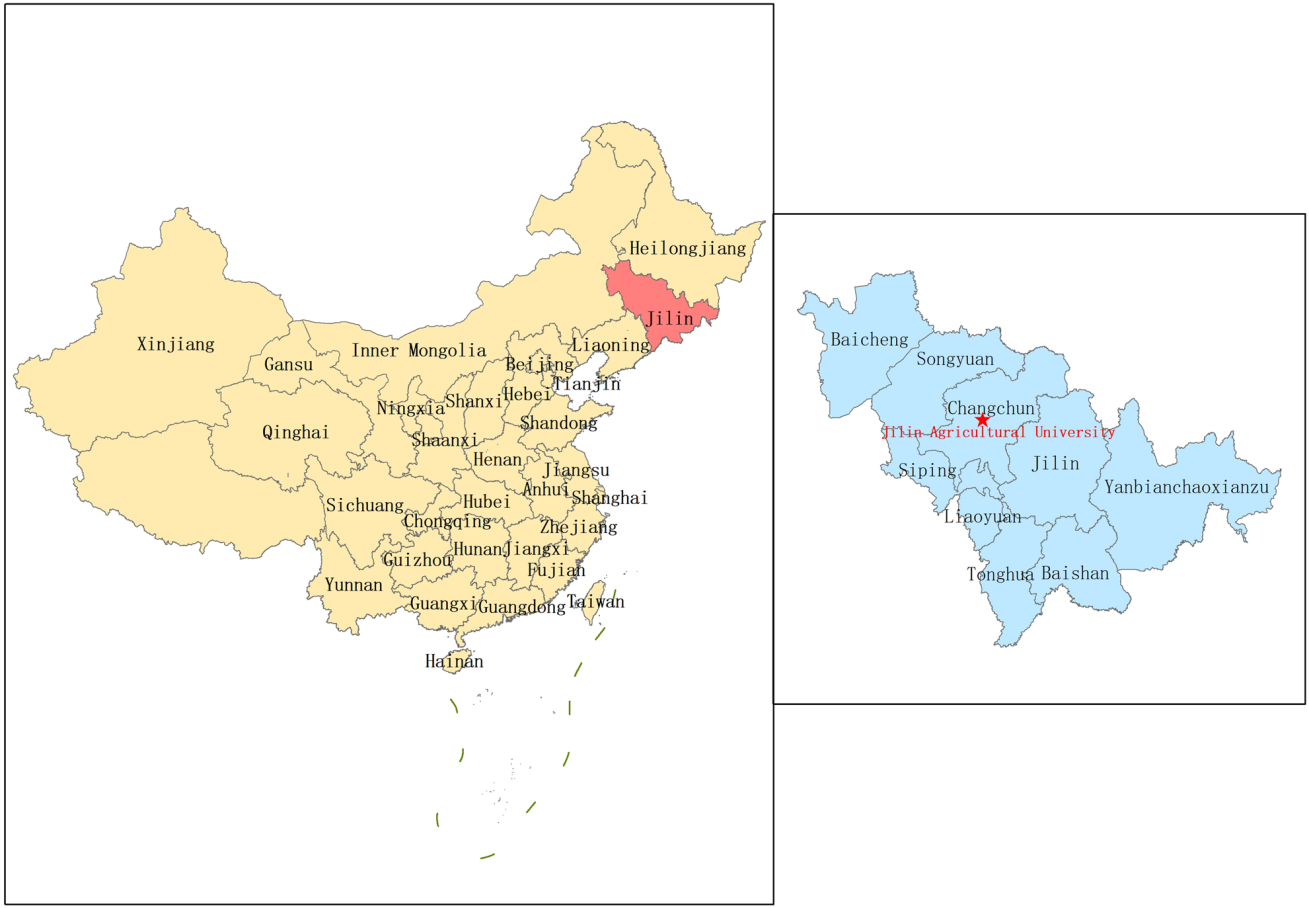
The pot experiment was conducted at Jilin Agricultural University in Jilin Province, China. The map is done using arcgis 10.8 (https://www.arcgis.com/index.html).

The pot experiment was conducted at Jilin Agricultural University in Changchun, Jilin Province, China (E 125°21 ′, N 43°52 ′) from April 2022 to October 2022, the specific location is shown in Fig. 1. The location has a temperate continental sub-humid monsoon climate with a sunshine duration of 2685 h. The accumulated temperature during the experiment ranged from 2840.1 to 3040 ℃ after reaching a minimum of 10 ℃. The frost-free period lasted for 145–150 days. The saline-sodic soil used in this study was obtained from Sheli Town, Da’an City, Jilin Province, China (N 45°35′58″-N 45°36′28″, E 123°50′27″-123°51′31″). The soil samples were collected, air-dried, and sieved through a 2 mm sieve. The table below (Table 1) provides the basic physical and chemical properties of the soil before the test. According to the World Soil Resources Reference Basis^29^.

**Table 1:** Basic physical and chemical properties of the tested soil.

Parameter	Unit	Mean
Bulk density	g cm^−3^	1.48
pH	–	9.44
Salinity	s·m^−1^	0.98
ENa^+^	mmol_e_·L^−1^	11.92
CEC	mmol_e_·L^−1^	13.98
ESP	%	48.8

### Experimental design

**Figure 2:**
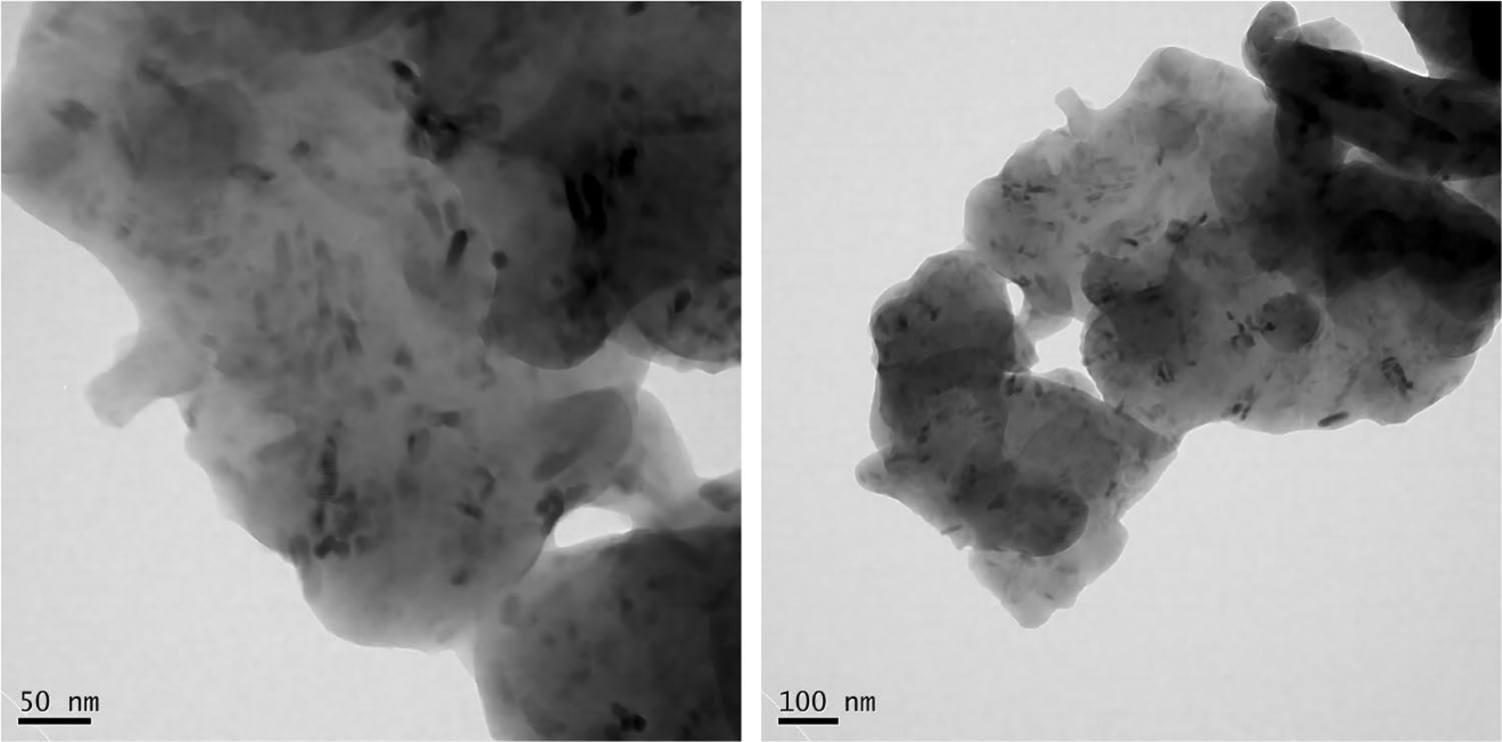
TEM scanning of ZnO NPs particles in deionized water.

The rice variety used in this experiment is Changbai 9, which is one of the excellent varieties cultivated in the saline-sodic paddy soil in northeast China. The pot planting method was employed, following a random block design. On April 12, 2022, rice seeds were sown in a greenhouse. Rice seedlings with similar growth were selected and transplanted into pots (upper diameter 30.50 cm, lower diameter 20.40 cm, height 26.20 cm, with 15.00 kg of soil in each pot) on May 21, 2022, at the three-leaf stage. Each pot contained three points, with three plants in each hole. There were a total of 25 pots in each treatment, and a total of three treatments. In this experiment, contrast (CT), ZnSO_4_ (Z), and ZnO NPs (nZ) were used. ZnSO_4_ heptahydrate at a concentration of 45 kg ha^−1^ was identified as the optimal concentration based on previous experiments. The ZnO NPs used in the study was sourced from Shanghai Chaowei Nano Technology Co., LTD, with an average particle size of 50 nm and a purity of 99.9%. The characterization structure of nanometer zinc oxide is shown in Fig. 2. The application amount of ZnO NPs was 30 kg ha^-1^. Both zinc sources were mixed with 5 cm of surface soil in the basin during transplanting. Detailed application amounts of ZnSO_4_ and ZnO NPs are shown in Table 2. The fertilizer dosage for each pot was calculated based on the field's recommended application rate (N 250 kg ha^−1^, P 100 kg ha^−1^, K 120 kg ha^−1^). The specific fertilizer dosages were as follows: urea 1.57 g pot^−1^, superphosphate 1.41 g pot^−1^, potassium sulfate 2.66 g pot^−1^. The first topdressing was done at the tillering stage with urea 1.18 g pot^−1^. The second topdressing was performed at the booting stage with urea 1.18 g pot^−1^ and potassium sulfate 2.66 g pot^−1^. The water level was maintained at a depth of 3–5 cm after transplanting and continued until two weeks before harvest. Insects, diseases, and grasses were strictly controlled throughout the rice growth period to prevent biomass and yield loss. The indexes were determined at the tillering stage.

**Table 2:** Application amounts of ZnSO_4_ and ZnO NPs.

Treatment	ZnSO_4_	ZnO NPs
Pot application amount (g pot^−1^)	0.81	0.54
Field application amount (kg ha^−1^)	45.00	30.00

### Sampling methods and measurements

#### Dry weight and Na^+^, K^+^ and Zn^2+^ content

The dry matter and the levels of Na^+^, K^+^, and Zn^2+^ in rice leaves were measured in rice plants that had uniform growth at the peak of tillering. For each treatment, 9 samples were taken from both above-ground and underground parts of the plants. These samples were dried at 105 ℃ for 0.5 h and then further dried at 80 ℃ until a constant weight was achieved. The dried leaf samples were ground into a fine powder and sifted. To determine the levels of Na^+^ and K^+^ in rice leaves, 0.500 g of accurately weighed sifted samples were digested using H_2_SO_4_-H_2_O_2_. The levels of Na^+^ and K^+^ in the plants were determined using a flame photometer (FP640, Shanghai Precision Technology Instrument Co., LTD.). To determine the content of Zn^2+^, a 0.1 g sample was accurately weighed using a 2 mm sieve. The weighed sample was then mixed with 3 mL of HNO_3_ and 1 mL of HF. Deboiling was carried out using a microwave digestion instrument (MD20H model, Chengdu Opole Instrument Co., LTD., China). After the sample became clear and transparent, the acid was removed using an electric graphite acid catcher (GD25/GD40, Chengdu Opole Instrument Co., LTD., China). The resulting solution was diluted to a constant volume of 100 mL using ultra-pure water. The content of Zn^2+^ in the plants was determined using an inductively coupled plasma emission spectrometer (ICP-1000II, Beijing Hao Micro Technology Co., LTD., China).

#### Content of malondialdehyde (MDA) and relative electrolyte leakage rate (REL) in rice leaves

The content of malondialdehyde (MDA) and relative electrolyte leakage rate (REL) in rice leaves were measured in rice plants at the peak of tillering. Malondialdehyde content was determined using thiobarbituric acid staining^30^, while REL in rice leaves was determined by Dionisiosese and Tobita (1998)^31^. To measure REL, fresh leaves (1.000 g) were washed with deionized water and placed in a test tube containing 15 ml of deionized water. After incubating for 2 h at room temperature (25 ℃), the conductivity (E1) was measured using a conductivity meter (DS-307, Shanghai Reitz, China). The test tube was then heated to 100 ℃ for 30 min and cooled to room temperature (25 ℃) to measure the electrical conductivity (E2). The relative electrolyte leakage rate (REL) was calculated using the following formula:

1 $$ {\text{REL}}\left( \% \right) = \frac{{{\text{E}}1}}{{{\text{E}}2}} \times 100. $$

#### Determination of chlorophyll content

At the peak of tillering, rice plants with uniform growth from each treatment were selected to determine the content of leaf pigment. Each treatment consisted of 9 holes. The first fully unfolded leaf from each hole of rice was taken, weighing 0.5 g, and mixed with ethanol, acetone, and water at a ratio of 4.5:4.5:1. The resulting mixture was then standardized to a volume of 25 ml. Absorbance values at 470, 645, and 663 nm were measured using an ultraviolet spectrophotometer (UV-2600, Shimadzu, Japan) to determine the pigment content. The calculation formula used is:

2 $$ {\text{Chlorophyll a}} = 12.21{\text{A}}_{663} - 2.81{\text{A}}_{645} , $$

3 $$ {\text{Chlorophyll b}} = {2}0.{\text{13A}}_{{{645}}} - {5}.0{\text{3A}}_{{{663}}} , $$

4 $$ {\text{Carotenoids}} = \, \left( {{1}000{\text{A}}_{{{47}0}} - {3}.{\text{27C}}_{{\text{a}}} - {1}0{\text{4C}}_{{\text{b}}} } \right) \, /{229,} $$

5 $$ {\text{Total chorophyll content}} = {\text{Chl a}} + {\text{Chl b}}{.} $$

#### Determination of gas exchange parameters

At the peak of tillering, the net photosynthetic rate (Pn), stomatal conductance (Gs), and intercellular carbon dioxide concentration of the first fully developed rice leaf were measured using the Li-6400, a portable photosynthesis measurement system by Li-Cor. The measurements were taken from 9:10 to 11:30 am during a sunny and windless tilling period. Each treatment was repeated 10 times to ensure accuracy. The leaf chamber temperature was maintained at approximately 26 ℃, and the light intensity was set at 800 μmol·m^-2^·s^-1^. Throughout the measurement process, the CO_2_ concentration was kept at 400 μmol·mol^-1^, and the relative humidity ranged between 60 and 70%.

#### Chlorophyll a fluorescence transient and 820 nm reflection

The chlorophyll fluorescence of rice with uniform growth in each treatment was measured at the peak of tillering, with 9 replicates per treatment. The leaves, which had uniformly grown, were dark adapted for 30 min in each treatment. The chlorophyll fluorescence fast induction kinetic curve (OJIP curve) were determined using M-PEA (Hansatech, UK). The OJIP curve was generated under 5000 μmol m^−2^·s^−1^ red light, with a measurement time of 2 s. The initial recording rate was 105 data per second^32^. The O point represents the fluorescence intensity at 0.02 ms, the K point represents the fluorescence intensity at 0.3 ms, the J point represents the fluorescence intensity at 2 ms, and the P point represents the maximum fluorescence, which is generally *F*_p_ ≈ *F*_m_. The OJIP curve is normalized to the OP point, determined by jip test analysis. The normalization is done using the formula *V*_t_ = (*F*_t_-*F*_O_)/(*F*_m_-*F*_O_), and the parameters and their meanings are shown in Table 3.

**Table 3:** Formulae and explanation the technical data of the OJIP curves and the selected JIP-test parameters used in this study.

Technical fluorescence parameters	
* F*_t_	Fluorescence at time t after onset of actinic illumination
* F*_o_ = *F*_20 µs_	Minimal fluorescence, when all PSII RCs are open
* F*_K_≡*F*_300 µs_	Fluorescence intensity at the K-step (300 µs) of OJIP
* F*_J_≡*F*_2 ms_	Fluorescence intensity at the J-step (2 ms) of OJIP
* F*_I_≡*F*_30 ms_	Fluorescence intensity at the I-step (30 ms) of OJIP
* F*_P_ (= *F*_m_)	Maximal recorded fluorescence intensity, at the peak P of OJIP
* V*_t_ = (*F*_t_-*F*_o_)/(*F*_m_-*F*_o_)	Relative variable fluorescence at time t
* W*_OI_ = (*F*_t_-*F*_o_)/(*F*_I_-*F*_o_)	Ratio of variable fluorescence *F*_t_-*F*_o_ to the amplitude *F*_I_-*F*_o_
* V*_J_ = (*F*_J_-*F*_o_)/(*F*_m_-*F*_o_)	Relative variable fluorescence at the J-step
* W*_k_ = (*F*_K_-*F*_o_)/(*F*_J_-*F*_o_)	Relative variable fluorescence at the K-step to the amplitude *F*_J_-*F*_o_
Quantum efficiencies or flux ratios	
_* φ*Po_ = TR_o_/ABS = 1-*F*_o_/*F*_m_	Maximum quantum yield for primary photochemistry
* Ψ*_Eo_ = ET_o_/TR_o_ = (1-*V*_J_)	Probability that an electron moves further than Q_A_
_* φ*Eo_ = ET_o_/ABS = (1-*F*_o_/*F*_M_) (1-*V*_J_)	Quantum yield for electron transport (ET)
_* φ*Do_ = 1-_*φ*Po_ = *F*_o_/*F*_m_	Quantum yield (at t = 0) of energy dissipation
_* φ*Ro_ = RE_o_/ABS = _*φ*Po_•*Ψ*_Eo_•_δRo_ = _*φ*Po_•(1-*V*_I_)	Quantum yield for reduction of the end electron acceptors at the PSI acceptor side (RE)
_ δRo_ = RE_o_/ET_o_ = (1-*V*_I_)/(1-*V*_J_)	Probability that an electron is transported from the reduced intersystem electron acceptors to the final electron acceptors of PSI (RE)
Specific energy fluxes and performance indexes	
M_0_ = 4(F_300μs–_F_o_)/(F_m–_F_o_)	Approximated initial slope (in ms) of the fluorescence transient normalized on the maximal variable fluorescence
ABS/RC = M_0_· (1–V_J_)· (1–*φ*_Po_)	Absorption flux per RC
TR_o_/RC = M_0_· (1–V_J_)	Trapped energy flux per RC (at t = 0)
ET_o_/RC = M_0_· (1–V_J_)·*Ψ*_Eo_	Electron transport flux per RC (at t = 0)
DI_o_/RC = (ABS/RC)—(TR_o_/RC)	Dissipated energy flux per RC (at t = 0)
RE_o_/RC = M_0_· (1–V_J_)·*Ψ*_Eo_·_*φ*Ro_	Reduction of end acceptors at PSI electron acceptor side per RC (at t = 0)
* PI*_ABS_ = $\left( {\frac{{{\text{RC}}}}{{{\text{ABS}}}}} \right)\left( {\frac{{\varphi_{{{\text{po}}}} }}{{1 - \varphi_{{{\text{po}}}} }}} \right)\left( {\frac{{\Psi_{{{\text{Eo}}}} }}{{1 - \Psi_{{{\text{Eo}}}} }}} \right)$	Performance index on absorption basis

#### Statistical analyses

All data were collected and analyzed using Microsoft Excel 2019 software. Subsequently, the data were analyzed using the SPSS statistical package version 22 (IBM Corp., Armonk, NY, USA). Descriptive statistics were employed to test the mean value and standard error of measurement parameters. One-way analysis of variance (ANOVA) was conducted in this study, and Duncan's multiple comparison method was utilized. The observed differences in comparisons were found to be statistically significant (p < 0.05). The results are presented as standard error (SE). The charts were generated using Origin 2021 software (https://www.originlab.com/2021).

### Ethics approval and consent to participate

All methods were performed in accordance with the relevant guidelines and regulations. We have obtained permission to collect plant material and seedlings.

## Results

### Plant growth

**Figure 3:**
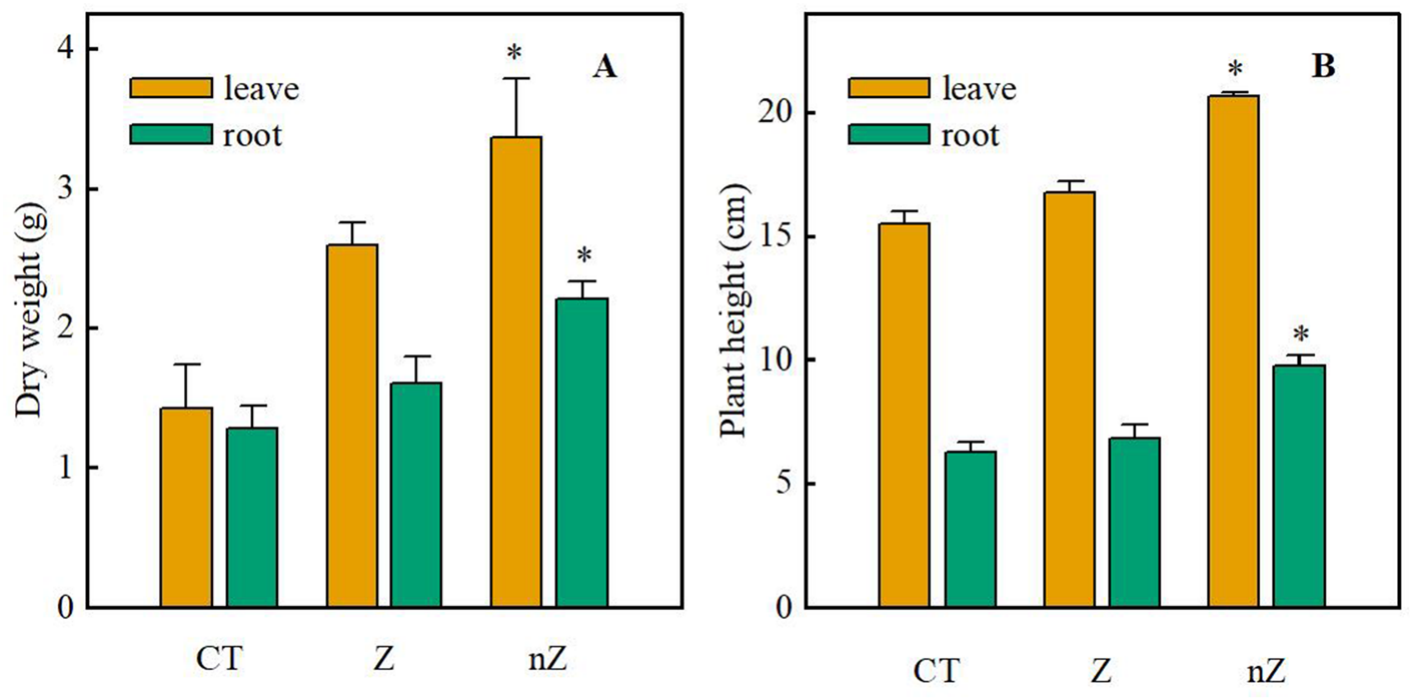
The effects of ZnO NPs on the dry weight (**A**) and plant height (**B**) of rice seedlings under saline-sodic stress. The mean values of nine repetitions ± SE (n = 9) were used, and different letters were used to indicate statistical significance at the p < 0.05 level. CT: saline-sodic soil, Z: saline-sodic soil applied with zinc sulfate, nZ: saline-sodic soil applied with nano-zinc oxide.

The effects of ZnSO_4_ and ZnO NPs on rice biomass are presented in Fig. 3A. The results indicate that both ZnSO_4_ and ZnO NPs significantly influenced the aboveground and root biomass of rice, which were significantly higher than the control group (CK). Moreover, the ZnO NPs treatment exhibited the most favorable results. Figure 3B shows the plant height of rice in saline-sodic land under the three treatments. The results demonstrate that both ZnSO_4_ treatment and ZnO NPs are beneficial for the growth of rice in saline-sodic land, with ZnO NPs showing a superior effect. These findings suggest that zinc can alleviate saline-sodic stress and promote the growth of rice in such soil, with ZnO NPs treatment being more effective in this regard.

### The concentration of Na^+^, K^+^, Zn^2+^, MDA and the relative electrical leakage in rice leaves

**Figure 4:**
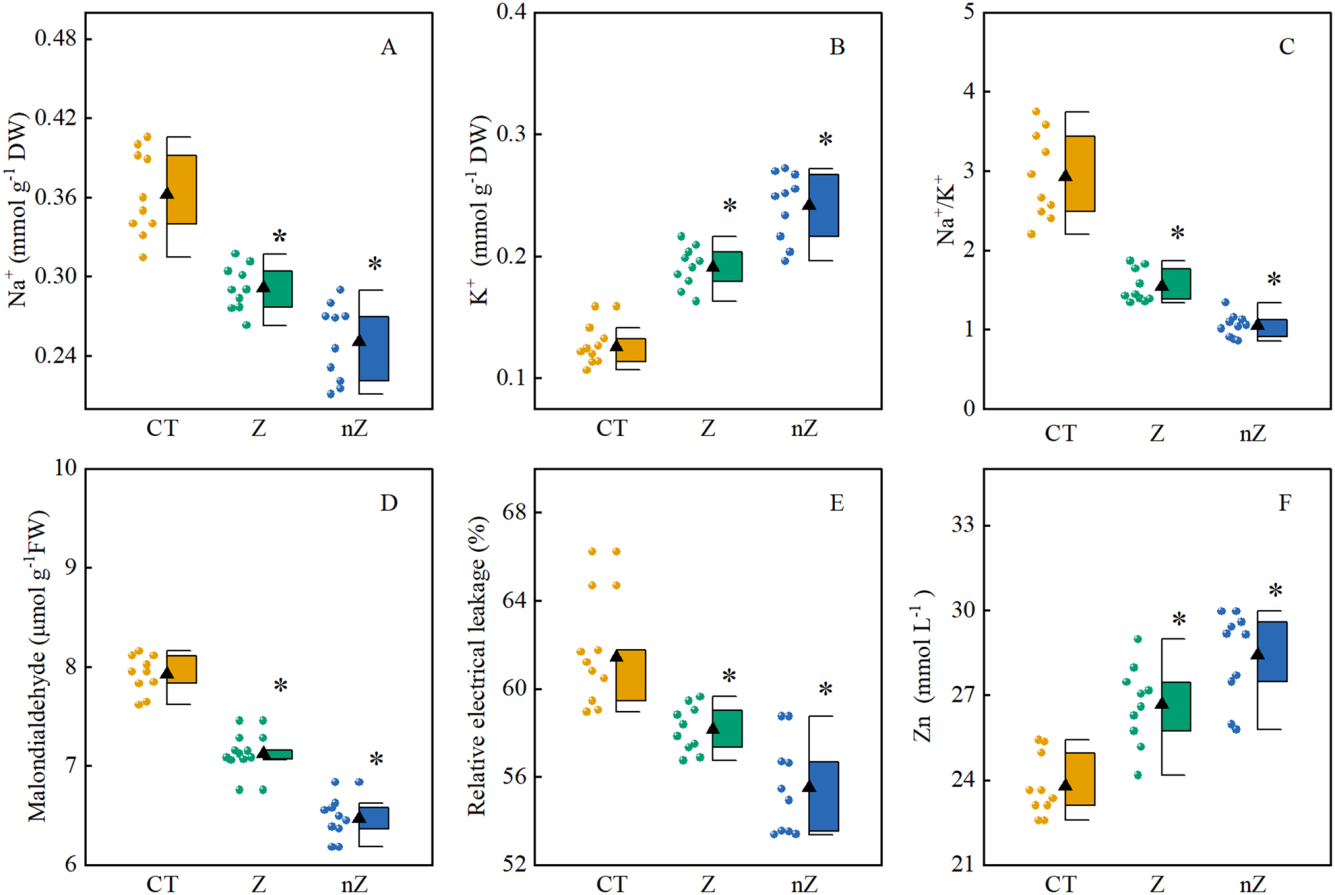
The effects of ZnO NPs on the concentration of Na^+^ (**A**), K^+^ (**B**), Na^+^/K^+^ (**C**), MDA (**D**), the relative electrical leakage (**E**) and Zn^2+^ (**F**), in rice leaves under saline-sodic stress. The mean ± SE (n = 9) of nine replicates was used, *: different letters were statistically significant at the p < 0.05 level. CT: Saline-sodic soil, Z: saline-sodic soil applied zinc sulfate, nZ: saline-sodic soil applied nano-zinc oxide.

The effects of ZnSO_4_ and ZnO NPs treatments on the concentrations of Na^+^, K^+^, and the Na^+^/K^+^ ratio in rice leaves in saline-sodic soil are shown in Fig. 4A,B. The results indicate that both ZnSO_4_ and ZnO NPs treatments led to a decrease in Na^+^ concentration (Fig. 4A) and the Na^+^/K^+^ ratio (Fig. 4B), while significantly increasing the K^+^ concentration (Fig. 4B) in the leaves. Among the treatments, ZnO NPs had the most pronounced effect on the Na^+^ and K^+^ concentrations and the Na^+^/K^+^ ratio in rice leaves from saline-sodic soil. Compared to the control (CK) and ZnSO_4_ treatments, the ZnO NPs treatment resulted in a significant decrease of 30.9 and 14.1% in the ratio of Na^+^ and Na^+^/K^+^ in rice leaves, respectively, and a significant increase of 91.6 and 26.2% in the concentration of K^+^.

Figure 4C illustrates the variation in zinc content in rice leaves grown in saline-sodic soil. The findings indicate that the application of exogenous zinc can notably enhance the zinc content in rice leaves. Moreover, the impact of ZnO NPs treatment on zinc content in rice leaves is significantly greater compared to ZnSO_4_ treatment. In comparison to the control group (CK) and ZnSO_4_ treatment, the ZnO NPs treatment resulted in a substantial increase of 19.6% and 6.6% in the zinc content of rice leaves, respectively.

Figure 4D,E illustrates the changes in malondialdehyde (MDA) content and relative electrolyte leakage rate (REL) in rice leaves from saline-sodic soil. The findings demonstrate that the application of exogenous zinc significantly reduces the MDA content and REL of rice leaves. Moreover, the effects of ZnO NPs treatment on MDA content and REL of rice leaves are even more pronounced. In comparison to the CK and ZnSO_4_ treatments, MDA content and REL are notably decreased by 18.4 and 9.2%, and 9.6 and 4.6%, respectively.

Figure 4F presents the correlation between leaf zinc content and malondialdehyde (MDA) content in rice leaves. The results indicate a negative correlation between zinc content in rice leaves from saline-sodic soil and MDA content in rice leaves (P < 0.01), with correlation coefficients of 0.74, respectively. These findings suggest a close relationship between the zinc content of rice leaves from saline-sodic soil and the content of malondialdehyde (MDA) in rice leaves.

### Chlorophyll and carotenoids

**Figure 5:**
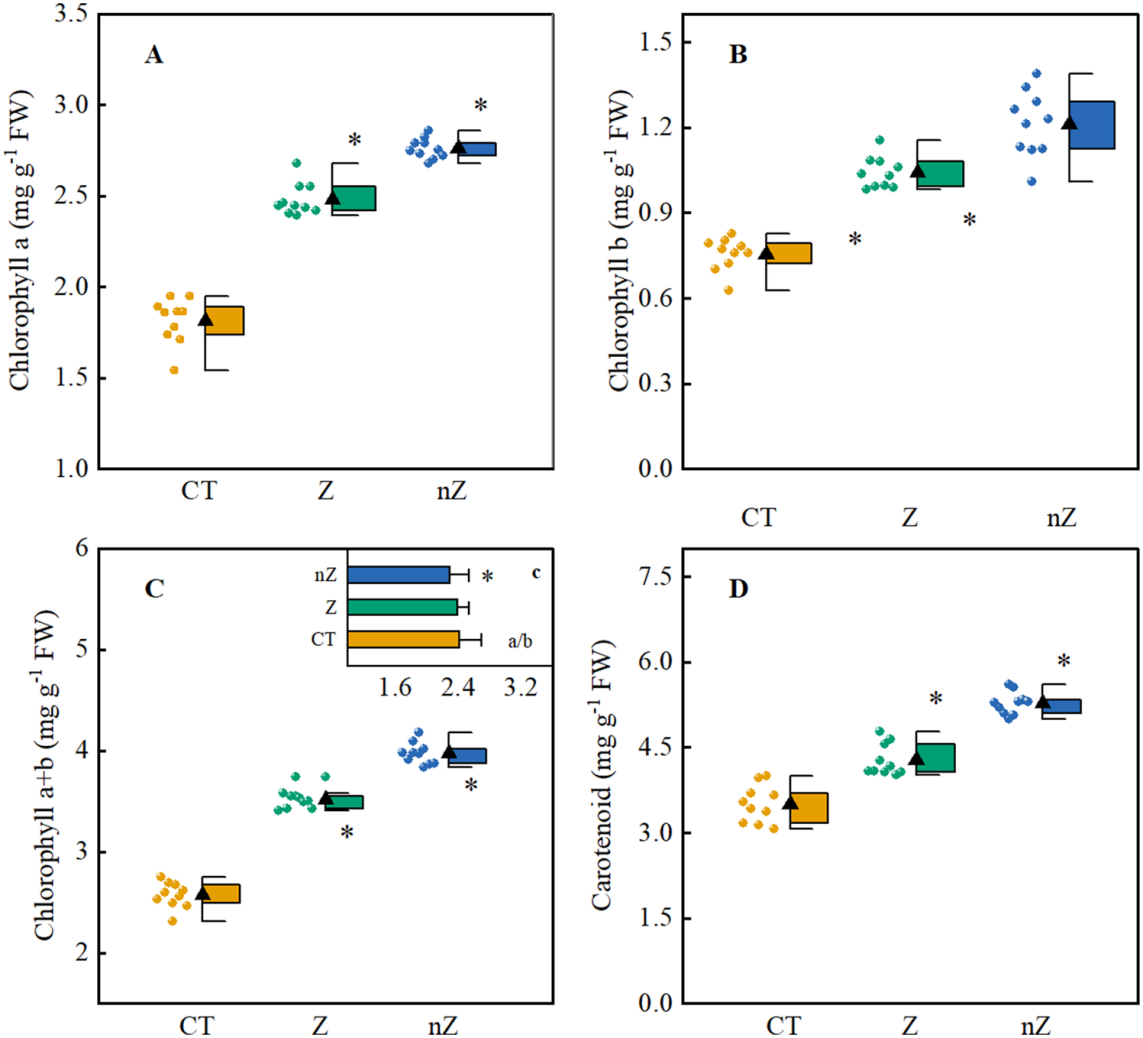
The effects of ZnO NPs on chlorophyll a (**A**), chlorophyll b (**B**), chlorophyll a + b (**C**), chlorophyll a/b (c) and carotenoid (**D**) contents in rice leaves under saline-sodic stress. The mean ± SE (n = 9) of nine replicates was used, *: different letters were statistically significant at the p < 0.05 level. CT: Saline-sodic soil, Z: saline-sodic soil applied zinc sulfate, nZ: saline-sodic soil applied nano-zinc oxide.

Figure 5 illustrates the changes in pigment content in rice leaves grown in saline-sodic soil. The results indicate that the application of exogenous zinc can effectively enhance the pigment content in rice leaves when subjected to saline-sodic stress. Furthermore, the treatment with ZnO NPs has a more pronounced effect on the levels of chlorophyll a, chlorophyll b, chlorophyll a + b, and carotenoids. Compared to the control group (CK), the ZnO NPs treatment significantly increased the content of chlorophyll a, chlorophyll b, chlorophyll a + b, and carotenoids in rice leaves by 52.1, 60.5, 54.6, and 22.7% respectively. Additionally, the application of ZnO NPs led to a significant decrease in the chlorophyll a/b value. Compared to the CK and ZnSO_4_ treatment, the ZnO NPs treatment resulted in a reduction of 5.0 and 3.8% in the chlorophyll a/b value of rice leaves. Therefore, the use of ZnO NPs is more beneficial for the synthesis of chloroplast pigments and helps mitigate the degradation of chlorophyll and carotenoids in rice leaves under saline-sodic stress.

### Gas exchange parameters

**Figure 6:**
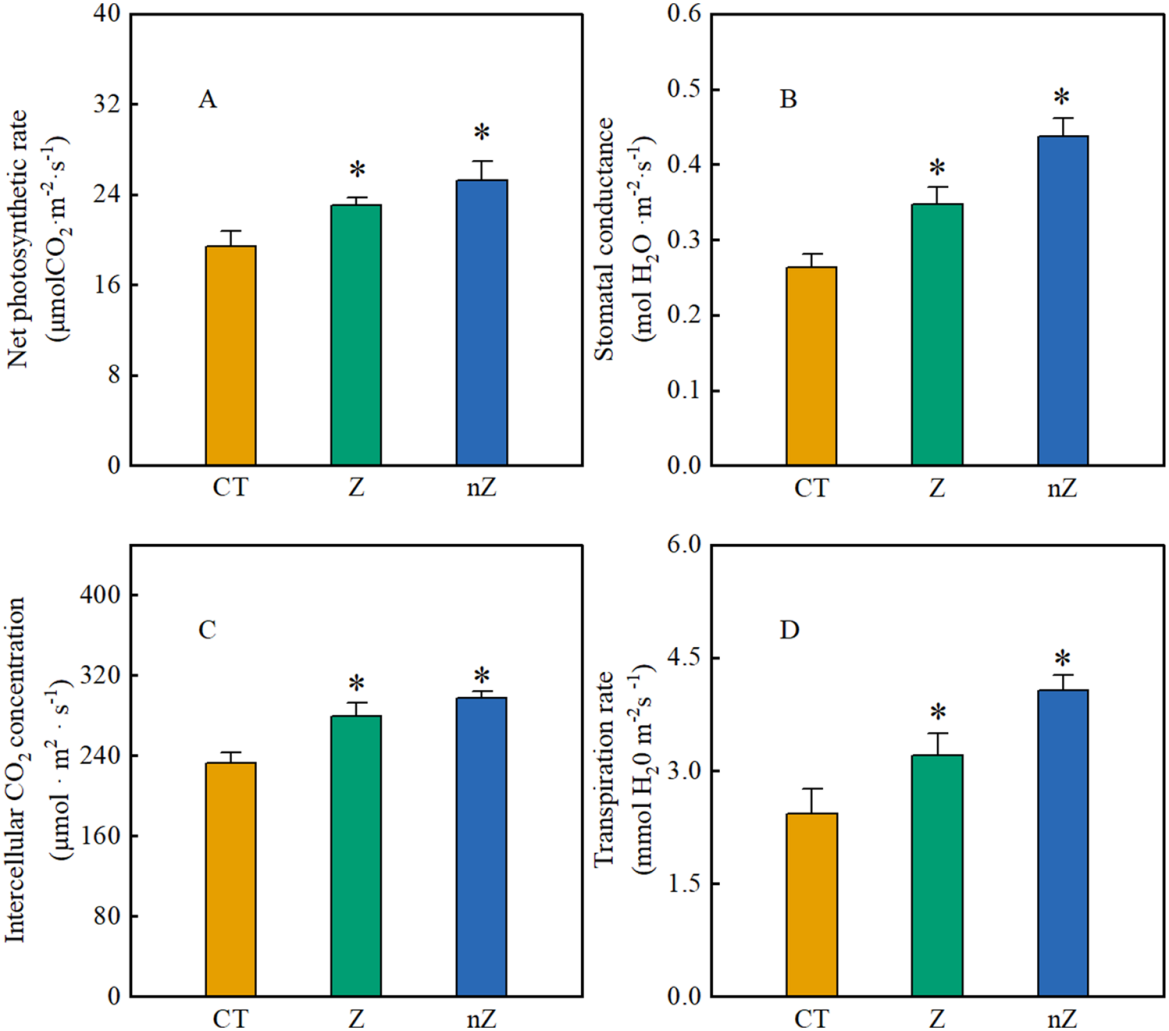
The effects of ZnO NPs on net photosynthetic rate (**A**), stomatal conductance (**B**), intercellular carbon dioxide concentration (**C**) and transpiration rate (**D**) in rice leaves under saline-sodic stress. The mean ± SE (n = 9) of nine replicates was used, *: different letters were statistically significant at the p < 0.05 level. CT: Saline-sodic soil, Z: saline-sodic soil applied zinc sulfate, nZ: saline-sodic soil applied nano-zinc oxide.

According to Fig. 6, when exposed to saline-sodic stress, exogenous zinc supplementation has been found to alleviate the stress and significantly enhance various parameters related to rice leaf photosynthesis. These parameters include the net photosynthetic rate, stomatal conductance, intercellular carbon dioxide concentration, and transpiration rate. Compared to the control group (CK), net photosynthetic rate of rice leaves treated with ZnSO_4_ and ZnO NPs increased by 19.1 and 30.5%, stomatal conductance increased by 38.7 and 46.8%, transpiration rate increased by 20.1 and 28.1%, and intercellular carbon dioxide concentration increased by 31.5 and 67.1%, respectively. Thus, zinc supplementation, particularly in the form of ZnO NPs, can effectively enhance the photosynthesis of rice leaves in saline-sodic soil.

### The O-P phase, *V*_J_, and* V*_I_ values

**Figure 7:**
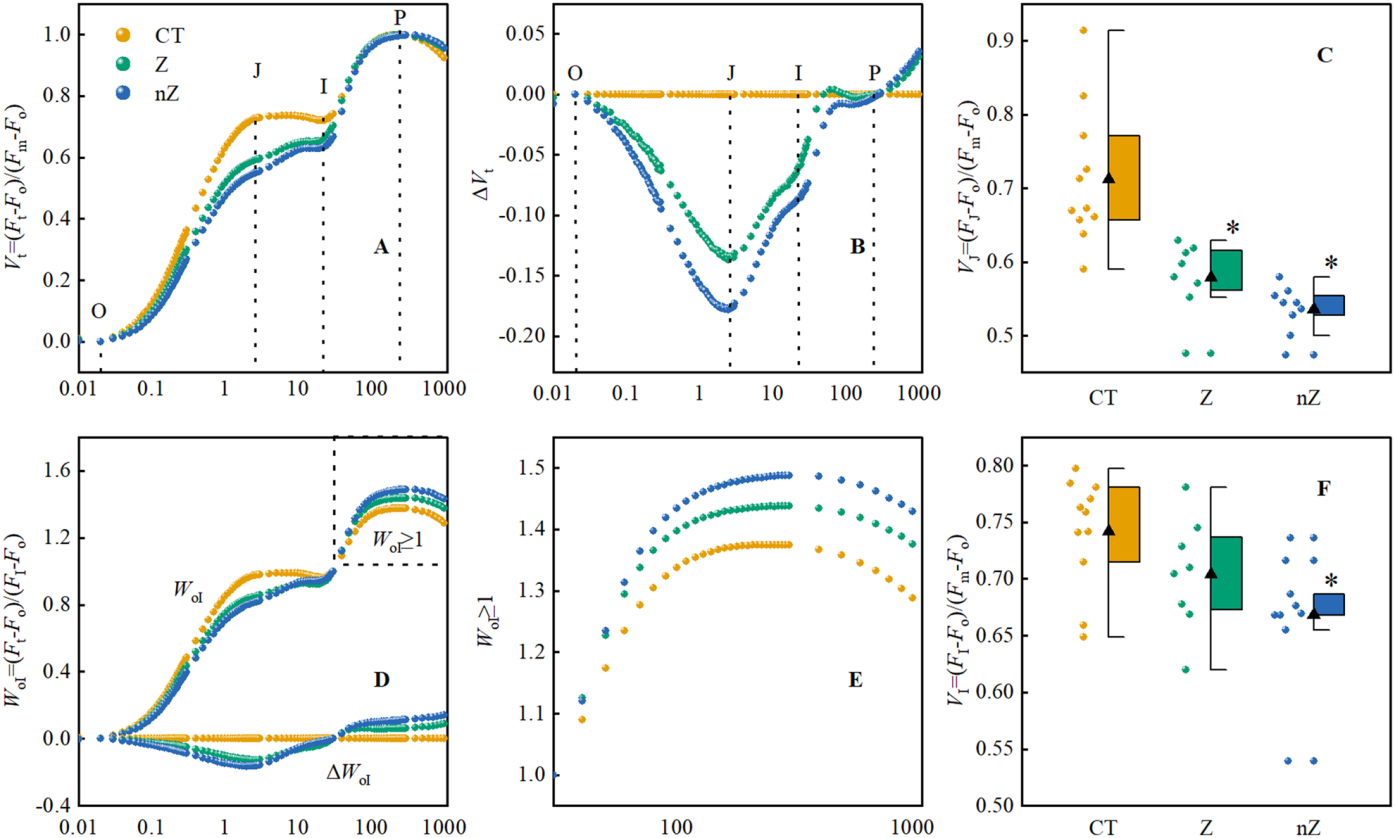
The effects of ZnO NPs on the relative variable chlorophyll fluorescence *V*_t_ and O-I phases of rice under saline-sodic stress. (**A, D**) shows the relatively variable *V*_t_ and O-I phases of chlorophyll fluorescence in rice leaves. **B, E** showed relatively variable chlorophyll fluorescence ∆*V*_t_ and W_OI_ ≥ 1 in rice leaves. (**C, F**) *V*_J_ and *V*_I_ are relatively variable fluorescence of step J and step I. The mean ± SE (n = 9) of nine replicates was used, *: different letters were statistically significant at the p < 0.05 level. CT: Saline-sodic soil, Z: saline-sodic soil applied zinc sulfate, nZ: saline-sodic soil applied nano-zinc oxide.

Figure 7A,B illustrates the chlorophyll fluorescence induction kinetics (*OJIP*) curve of rice. When ZnSO_4_ and ZnO NPs were applied under saline-sodic stress, the J and I points on the *OJIP* curve of rice leaves decreased compared to the control (CT). Figure 7B shows that the decline in ∆*V*_t_ was the largest under ZnO NPs treatment. And, under ZnO NPs treatment, ∆*V*_t_ had the lowest value at J and I. The amplitude of phase I-P in the *W*_OI_ ≥ 1 reflects the size of the end electron acceptor pool on the PSI acceptor side, with a smaller amplitude indicating a smaller end electron acceptor pool on the PSI acceptor side. The O-I (Fig. 7D,E) phase analysis revealed that the application of ZnO NPs under saline-sodic stress had the most significant effect on improving the terminal electron acceptor pool on the PSI acceptor side of rice leaves. Additionally, we calculated *V*_J_ and *V*_I_ values (Fig. 7C,F), and the results indicated that compared to CT (control treatment), the *V*_J_ and *V*_I_ values of rice leaves significantly decreased when ZnSO_4_ and ZnO NPs were applied under saline-sodic stress. Specifically, the values of *V*_J_ and *V*_I_ were reduced by 24.8% and 9.9% respectively when treated with ZnO NPs, compared to CT and ZnSO_4_ treatment which showed reductions of 7.5 and 5.1% respectively. Therefore, under saline-sodic stress conditions, the application of ZnO NPs not only benefits the performance of PSII on the donor side (K-step reduction) and acceptor side (J-step reduction), but also effectively enhances the size of the electron acceptor pool at the PSI acceptor end.

### The JIP parameters estimating the quantum yields, efficiencies and probabilities

**Figure 8:**
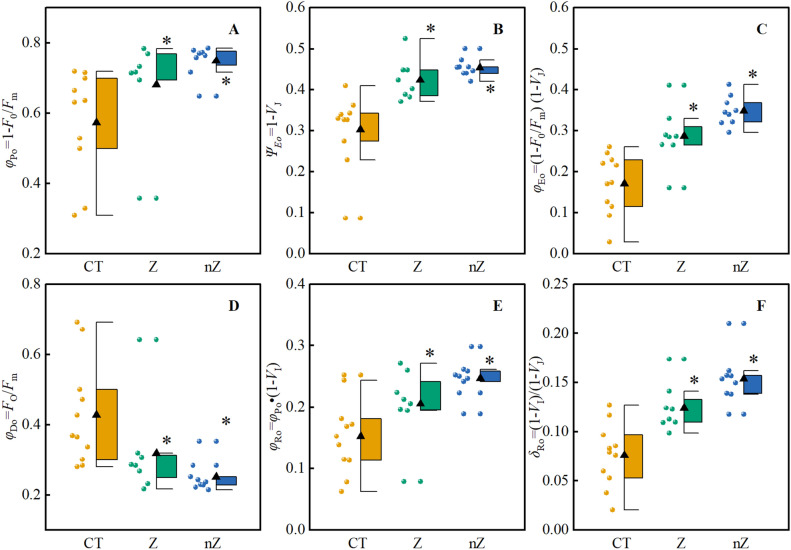
Effects of ZnO NPs on JIP parameters of quantum yield, efficiency, probability and electron transport of rice under saline-sodic stress. *φ*_Po_ (**A**), *ψ*_EO_ (**B**), *φ*_Eo_ (**C**), *φ*_Do_ (**D**), *φ*_Ro_ (**E**) and *δ*_Ro_ (**F**) refer to Table 2. The mean ± SE (n = 9) of nine replicates was used, *: different letters were statistically significant at the p < 0.05 level. CT: Saline-sodic soil, Z: saline-sodic soil applied zinc sulfate, nZ: saline-sodic soil applied nano-zinc oxide.

Figure 8 presents the effective parameters, such as the quantum yield of rice seedlings, under saline-sodic stress. The application of ZnSO_4_ and ZnO NPs significantly increased the values of *φ*_Po_, *ψ*_Eo_, *φ*_Eo,_ *φ*_Ro_ and *δ*_Ro_ in rice leaves, while the values of *φ*_Do_ decreased significantly. Notably, the treatment with ZnO NPs showed the most significant improvement. Compared to the control group (CT), the values of *φ*_Po_, *ψ*_Eo_, and *φ*_Ro_ increased by 30.7, 49.8, and 61.8%, respectively, while the value of *φ*_Do_ decreased by 41.2%. These findings suggest that the use of ZnO NPs is more advantageous in enhancing the quantum yield and efficiency of rice under saline-sodic stress. It also reduces the energy dissipation ratio and improves the photosynthetic fluorescence performance index.

### The ***PI***_ABS_ and specific energy flux for each PSII active reaction center

**Figure 9:**
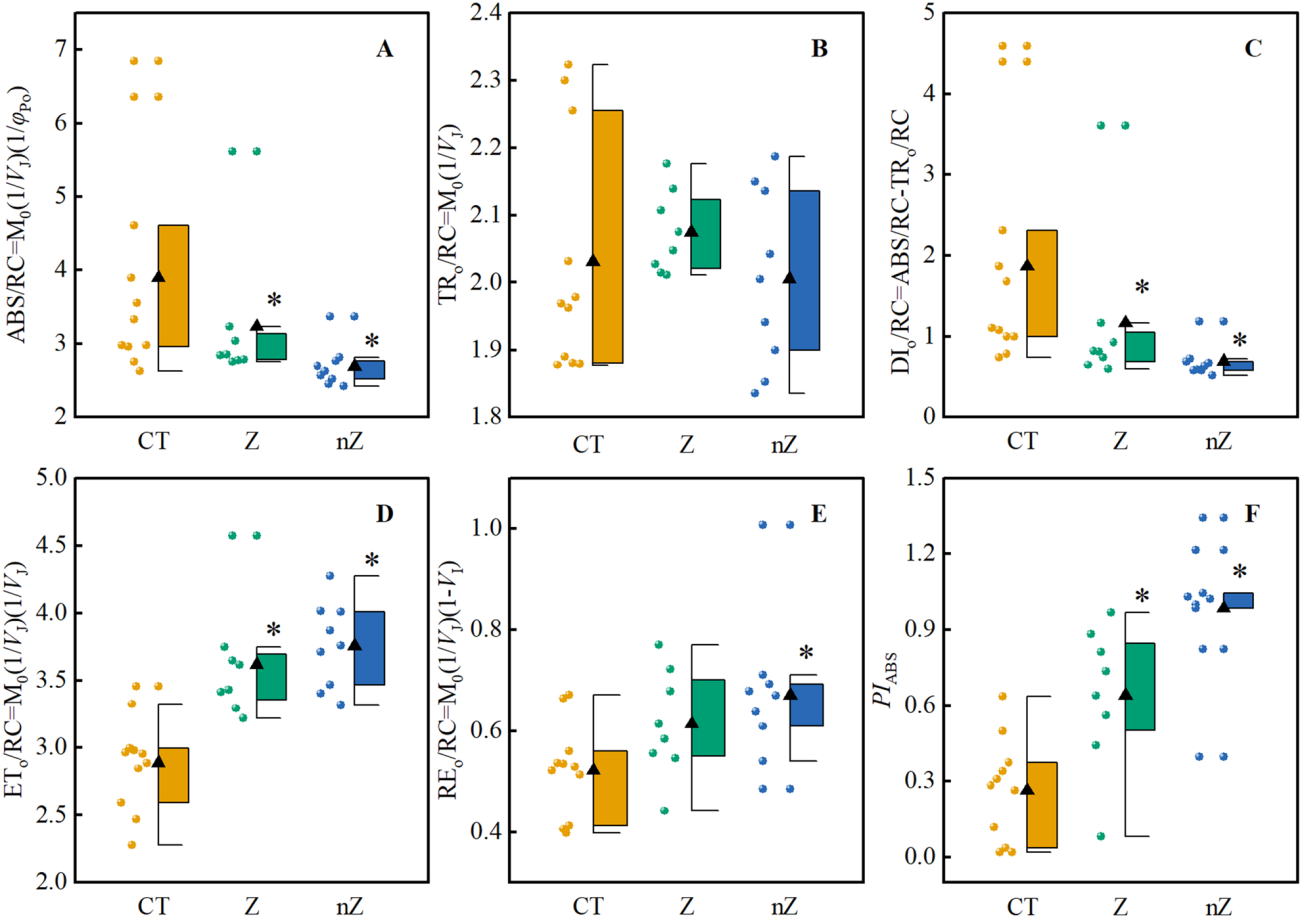
Effect of ZnO NPs on specific energy flux of rice PSII reaction center under saline-sodic stress. ABS/RC (**A**), TR_o_/RC (**B**), DI_o_/RC (**C**), ET_o_/RC (**D**), RE_o_/RC (**E**) and fluorescence performance index (**F**).The mean ± SE (n = 9) of nine replicates was used, *: different letters were statistically significant at the p < 0.05 level. CT: Saline-sodic soil, Z: saline-sodic soil applied zinc sulfate, nZ: saline-sodic soil applied nano-zinc oxide.

**Figure 10:**
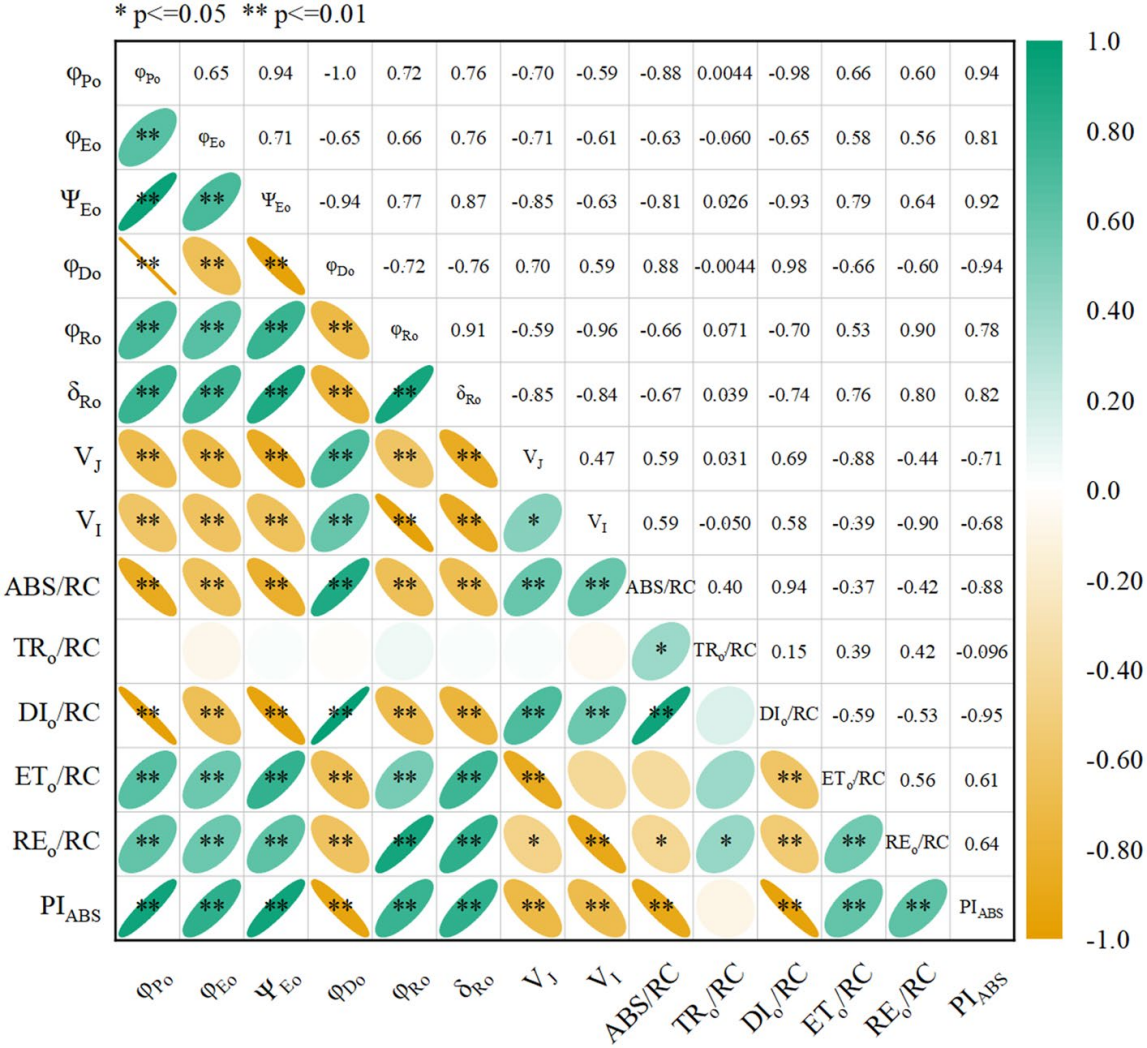
Correlation analysis of *PI*_ABS_, *φ*_Po_, *ψ*_Eo_, *φ*_Eo_, *φ*_Do_, *φ*_Ro_, *δ*_Ro_, *V*_J_, *V*_I_, ABS/RC, TR_o_/RC, DI_o_/RC, ET_o_/RC and RE_o_/RC. * was significantly correlated with P < 0.05, ** was significantly correlated with P < 0.01.

The results in Fig. 9F demonstrate that the application of ZnSO_4_ and ZnO NPs significantly increased the *PI*_ABS_ index of rice leaves under saline-sodic stress. Additionally, the study revealed that ZnSO_4_ and ZnO NPs application reduced the ABS/RC and DI_o_/RC values (Fig. 9A,C), while increasing the ET_o_/RC and RE_o_/RC values (Fig. 9D,E) under saline-sodic stress, and it has no significant impact on TRo/RC (Fig. 9B). Notably, ZnO NPs exhibited the most favorable effect. These findings indicate that, under saline-sodic stress, a greater amount of energy is absorbed by the unit reaction center for trapping and heat dissipation, with less energy being transferred downstream. However, the application of ZnO NPs positively influenced the absorption, transfer, and transfer of energy in the reaction center of rice leaves under saline-sodic stress. Figure 10 further illustrates the positive correlation between *PI*_ABS_ and *φ*_Po_, *ψ*_Eo_, *φ*_Eo_, *φ*_Ro_, *δ*_Ro_, ET_o_/RC, and RE_o_/RC. Conversely, *PI*_ABS_ exhibited negative correlations with *V*_J_, *V*_I_, *φ*_Do_, ABS/RC, and DI_o_/RC.

## Discussion

Saline-sodic soil has a significant impact on plant growth and development^33^. This is mainly caused by the high concentration of Na^+^ in the soil, which leads to excessive absorption of Na^+^ by plants and inhibits the absorption of essential nutrient ions such as K^+^, Zn^2+^, and Fe^3+^. Such disturbance can have negative effects on plant growth, development, and even survival^34^. Research has shown that the excessive accumulation of Na^+^ in plant tissues disrupts the integrity and function of cell membrane structures. This disruption leads to an imbalance of reactive oxygen species (ROS) and leads to an increase in MDA content, which increases the relative electricity leakage (REL) of plant tissues^35^. In this study, it was observed that the application of zinc promoted the uptake of Zn^2+^ in rice, enhancing its ability to resist saline-sodic. Zinc not only reduced the absorption of Na^+^ and increased the absorption of K^+^, but also inhibited the production of MDA and reduced REL, thereby maintaining the integrity of the cell membrane^36^. As can be seen in Fig. 4F, there was a significant negative correlation between zinc content and malondialdehyde (MDA) content in rice leaves in saline-sodic land. In recent years, the green synthesis of nanoparticles has attracted much attention because of its advantages of low cost, simplicity and environmental protection^37^. This study found that ZnO NPs was more conducive to the absorption of zinc in rice and had a better alleviation effect on saline-sodic stress. This may be due to the fact that nanoparticles can create higher surface area utilization, which allows rice plants to absorb higher tissue Zn content^38^.

Chlorophyll is a vital substance for photosynthesis in plants as it is closely involved in the absorption and conversion of light energy^39^. The findings of this study revealed that a high pH level reduces the availability of important nutrients such as Zn^2+^ and Mg^2+^ and inhibits chlorophyll synthesis under saline-sodic stress conditions. The combination of saline-sodic stress leads to ion stress and osmotic stress on rice, resulting in the generation of reactive oxygen species that damage the structure of chloroplasts and accelerate the degradation of rice leaf pigments^40^. Rice plants can counteract photoinhibition by adjusting the ratio of leaf chlorophyll to reduce the amount of captured light energy, thereby facilitating growth throughout the entire growth period^4^. In this study, the application of ZnO NPs zinc significantly increased the contents of chlorophyll a and chlorophyll b in rice leaves, resulting in a decrease in the chlorophyll a/b ratio. The chlorophyll a/b ratio serves as an indicator of plant stress resistance and exhibits a negative correlation with photosynthetic efficiency^41^. Hence, the reduced a/b value due to ZnO NPs application suggests that it enhances the ability of rice to alleviate saline-sodic stress. Furthermore, the application of ZnO NPs also significantly enhances the gas exchange parameters of rice leaves, with the variation in Pn (net photosynthetic rate) being consistent with the intercellular CO_2_ concentration^42^. This suggests that plant photosynthesis is influenced by stomatal factors. The similarity in the hydration radius of Na^+^ and K^+^ under saline-sodic stress creates intense competition between Na^+^ and K^+^ channels, which inhibits K^+^ absorption^43^. However, the application of ZnO NPs inhibits Na^+^ absorption and promotes K^+^ absorption, thereby improving stomatal conductance in rice leaves, preserving cell membrane integrity, and creating a favorable environment for photosynthesis, ultimately enhancing the gas exchange parameters of rice.

The results indicated that the application of ZnO NPs enhanced the growth and physiological properties of crops experiencing stress. This can be attributed to the high affinity of nanoparticles for absorption by plants, thereby promoting crop growth and development^44^. A study was conducted to investigate the impact of ZnO NPs on chlorophyll fluorescence in rice leaves under saline-sodic stress using rapid chlorophyll fluorescence kinetic technique and jep-text analysis^45^. Chlorophyll, as a key light-absorbing molecule, provides valuable insights into the structure, conformation, and function of photosynthetic devices through its fluorescence^46^. Plant leaves exhibited distinct *OJIP* transients under continuous light, representing the multiphase curve induced by chlorophyll fluorescence. The chlorophyll fluorescence gradually increased from the initial fluorescence point O to the maximum fluorescence point P, reflecting the fluorescence kinetic process of *OJIP*^47^. Rapid chlorophyll fluorescence ascent kinetics typically involve multiple stages, namely O (20 ms, RCS all on), J (2 86 ms), I (30 ms), and P (equal to Fm, RCS all off). Monitoring chlorophyll fluorescence is a widely used method to assess the photosynthetic performance of plants. This technique enables the observation of changes in chlorophyll fluorescence, which in turn provides information about the stability and efficiency of thylakoid membranes^47^. Previous studies have demonstrated that under saline-sodic stress, chlorophyll degradation occurs, leading to the disruption of donor/acceptor side performance and reaction center^48^. Consequently, the photochemical activity of PSII is reduced, and the electron transport ability is inhibited, resulting in an overall decrease in PSII performance and a decline in the photosynthetic ability of rice leaves. The results of this study demonstrated that the application of ZnO NPs caused changes in the fluorescence values of each phase on the curve, with the J-point (*V*_J_) being the most affected. This indicates that the performance of the PSII receptor side was impaired, leading to the destruction of the D_1_ protein on the receptor side and hindrance in the electron transfer from Q_A_ to Q_B_. The quantum yield and flux ratios *φ*_Po_, *φ*_Eo_, *ψ*_Eo_, *φ*_Do_ reflect the allocation of energy in plant absorption, capture, transfer, and heat dissipation^49^. The results of this experiment revealed that under saline-sodic stress, ZnO NPs increased the values of *φ*_Po_, *φ*_Eo_, *ψ*_Eo_, and decreased the value of *φ*_Do_ in rice leaves.The decrease in photochemical reaction efficiency (*φ*_Po_) under saline-sodic stress inevitably results in increased energy dissipation, including heat, fluorescence, and energy transfer to other systems. The application of ZnO NPs reduces energy dissipation, increases *φ*_Po_, and decreases *φ*_Do_^50^. This highlights the significance of Zn in the electron transport chain, and an optimal increase in its content enhances the effectiveness of the electron acceptor and facilitates electron transport between PSI and PSII.

The performance index *PI*_ABS_ is a measure of the overall state of the plant's photosynthetic apparatus^51^. It effectively indicates the extent of damage to the PS II of plant leaves under saline-sodic stress. Previous studies have demonstrated that the *PI*_ABS_ of wheat leaves decreases when exposed to saline-sodic stress, primarily due to osmotic stress and ion stress^52^. In this study, the application of ZnO NPs improved the electron transfer efficiency and photochemical activity of PSII, resulting in an overall improvement in the performance of PSII *PI*_ABS_ in rice leaves and promoting normal photosynthesis. This improvement may be attributed to the reduction of the *V*_J_ value by ZnO NPs (Fig. 7C). Other studies have indicated that an increase in *V*_J_ is associated with the damage level of PSII donor and acceptor sides under saline-sodic stress^53^. The reduction of ZnO NPs application improves the damage level of PSII donor and acceptor sides, thereby contributing to the increase in *PI*_ABS_^54^. Furthermore, the application of ZnO NPs was found to significantly reduce ABS/RC and increase ETo/RC and REo/RC in rice^55^. This suggests that ZnO NPs application enhances the light harvesting efficiency and transfer efficiency of rice, further supporting the increase in *PI*_ABS_.

Saline-sodic stress has been found to have a negative impact on chlorophyll synthesis and the electron transport chain between PSI and PSII^55^. Zinc not only reduces the absorption of Na^+^ by rice, thereby alleviating saline-sodic stress, but also aids in the synthesis of chlorophyll. This creates an optimal environment for photosynthetic electron transfer, effectively promoting electron transfer between PSI and PSII^56^. Compared to zinc sulfate, ZnO NPs demonstrate a more favorable effect. ZnO NPs are widely utilized in various fields. The unique nanostructure and nanoproperties of ZnO NPs have garnered significant attention from scientists^38^. In the realm of agricultural production, these nanoparticles have demonstrated promising effects, including the promotion of seed germination and seedling growth, mitigation of abiotic stress, and enhancement of plant resistance. Nevertheless, it is crucial to acknowledge the potential negative impacts of ZnO NPs, as several studies have indicated a dose-dependent effect^57^. High doses of ZnO NPs can hinder plant growth by inhibiting germination and chlorophyll biosynthesis, leading to reduced biomass accumulation and ultimately affecting crop development^58^. Therefore, the application of ZnO NPs should be carefully managed to ensure optimal utilization.

## Conclusion

Compared to ZnSO_4_, the use of ZnO NPs has been found to be more beneficial in enhancing the growth and physiological characteristics of rice in saline-sodic environments. The application of ZnO NPs not only hinders the absorption of Na, thereby reducing the detrimental effects of saline-sodic stress on rice, but also facilitates the absorption of Zn, leading to an increase in the chloroplast pigment content of rice under saline-sodic stress and promoting efficient photosynthesis. Additionally, the application of ZnO NPs enhances the performance of the electron acceptor side of the electron transport chain, thereby facilitating electron transfer between PSII and PSI. In conclusion, this study emphasizes the significance of zinc oxide nanoparticles in improving the growth and photosynthetic efficiency of rice in saline-sodic soils.

## Data Availability

The data that support the findings of this study are available on request from the corresponding author.
